# An Experimental and Numerical Analysis of Glued Laminated Beams Strengthened by Pre-Stressed Basalt Fibre-Reinforced Polymer Bars

**DOI:** 10.3390/ma16072776

**Published:** 2023-03-30

**Authors:** Agnieszka Wdowiak-Postulak, František Bahleda, Jozef Prokop

**Affiliations:** 1 Faculty of Civil Engineering and Architecture, Kielce University of Technology, 25-314 Kielce, Poland; 2 Faculty of Civil Engineering, University of Zilina, Univerzitná 8215/1, 010 26 Zilina, Slovakia

**Keywords:** timber beams, strengthening, near surface mounted (NSM), bars, BFRP, bending resistance

## Abstract

Damage often develops in glued laminated timber members under high bending loads due to natural defects in the timber, which results in their low load-bearing capacity and stiffness. In order to improve the bending mechanical properties of glulam beams, a new type of longitudinal glulam reinforcement with pre-stressed basalt fibre-reinforced polymer composites (BFRP) was developed using the Near Surface Mounted (NSM) technique. The strengthening method consisted of two pre-stressed BFRP bars glued into the grooves at the bottom side of the beam; meanwhile, for the second strengthening alternative, the third BFRP bar was embedded into the groove at the top side of the beam. Therefore, an experimental study was carried out to verify this strengthening technique, in which fifteen full-size timber beams were tested with and without bonded BFRP bar reinforcement in three series. According to the results of this experimental study, it can be seen that the effective load-bearing capacity of the reinforced beams increased up to 36% and that the stiffness of the beams increased by 23% compared to the unreinforced beams. The tensile stresses in the wooden fibres were reduced by 11.32% and 25.42% on average for the beams reinforced with two and three BFRP bars, respectively. On the other hand, the compressive stresses were reduced by 16.53% and 32.10% compared to the unreinforced beams. The usual failure mode saw the cracking of the wood fibres at the defects, while for some specimens, there were also signs of cracks in the epoxy adhesive bond; however, the crack propagation was, overall, significantly reduced. The numerical calculations also show a good correlation with the experimental results. The difference in the results between the experimental and numerical analysis of the reinforced and unreinforced full-sized beams ranged between 3.63% and 11.45%.

## 1. Introduction

The load-carrying capacity of timber structures can be significantly lowered due to the action of various factors. However, various methods can be applied in order to strengthen them depending on the application of the strengthened structure [1,2]. In the case of members under flexural stress, due to the ability of timber to resist both compressive and tensile stress, strengthening in both regions can be considered. Nowadays, the most common method of strengthening timber in the compression zone is by connecting the timber beam with a new compressed concrete slab. The connection can be carried out via several methods, either by screwed or adhesive connections [3,4,5]. A more traditional method of strengthening timber beams involves using steel and aluminium plates or bars in the tension zone. With the development of new progressive materials, however, the possibility of replacing steel or aluminium with more eco-friendly materials, such as FRP composites, could be achieved. Strengthening timber beams using steel or FRP materials in the tension zone is a very effective method of improving the resistance and stiffness of timber structures [1,6,7]. In addition, the use of plant-based fibres for making sustainable eco-friendly and biodegradable materials has increased. Fibres from plants such as a cotton, hemp, pineapple, etc., can be used as reinforcements in polymer matrix composites alone, as well as in combination with common synthetic fibres [8,9]. However, it is important to note that, in comparison to steel, the use of FRP as a reinforcement, can provide better properties, such as a higher strength-to-weight ratio and better corrosion resistance [1,10]. When comparing the synthetic FRP materials to each other, every material provides different advantages. CFRPs have better mechanical properties, such as a higher tensile strength and a significantly higher tensile modulus of elasticity; however, their price is also several times higher. In addition, their fatigue and creep resistance are better than those of GFRPs and BFRPs. On the other hand, the elongation at rupture of GFRP and BFRP materials is higher and the manufacture of these two types of fibre composites is more environmentally efficient; indeed, this is becoming an important feature in nearly every kind of industry. Therefore, the material selection for strengthening is dependent on the convenient utilization of the material properties and user preference.

Reinforcements in the form of FRP composite materials are used to repair, rehabilitate, retrofit, or strengthen building structures [1,11,12]. The primary methods of reinforcing timber structures using FRP composites involve either external or internal systems or bonded systems. For example, considering external reinforcement (e.g., EBR), FRP sheets or fabrics are used in the tension zone attached to the element’s surface [12,13,14,15]. Internally bonded systems, such as rods or sheets, are usually glued into grooves or slots of timber members; this type of reinforcement is referred to as near-surface mounted reinforcement, or NSM. Due to the greater interfacial area between the main structural material and the adhesive materials, NSM has greater load-bearing capacity and bonding resistance [16,17].

Recently, fibre-reinforced polymers (FRPs) have been used in the reinforcement of structural timber [18,19]. Wood reinforcement using FRP and adhesives is an important issue in the design of engineering structures [20,21,22,23,24,25,26,27,28,29,30,31,32,33,34,35]. Moreover, this bonding is considered to be the best method for stress transfer between FRP material and wood [36]. These conclusions are related to the elimination of stress concentrations in mechanical fasteners (e.g., nails, screws or dowels). Therefore, a better composite interaction between the materials can be achieved, as the stresses are uniformly distributed both along the base structural material and the adhesive [37]. On the other hand, it has to be mentioned that the glass fibre reinforced polymer (GFRP) is currently the most widely used type of FRP material, due to its greater cost-effectiveness [38]. In addition, a newer type of FRP is basalt fibre-reinforced polymer rods (BFRP) [18]. The basalt is also known as solidified volcanic lava, which is an effusive rock formed when molten lava pours out of the depths of the earth and hardens [39]. Therefore, the basalt can be considered as a renewable and sustainable resource material [40].

BFRP bars have a high ultimate tensile strength, ranging from 920 to 1650 MPa, as well as a relatively low modulus of elasticity, ranging from 45 to 59 GPa [41]. These properties are primarily relevant when used in pre-stressed concrete, as its strength is comparable to the strength of traditional pre-stressing steel; simultaneously, it has modulus of elasticity that is approximately four times lower. Thus, both elastic and long-term pre-stressing losses in pre-stressed concrete elements due to the shortening of the bar can be significantly reduced.

An experimental study of glued-in bars can also be found in the work of [42], and studies have also been carried out on the use of BFRP bars in concrete structures, primarily in bridge applications [43]. After reviewing the literature, it was found that the amount of experimental research using the NSM technique in timber structures is still relatively small [18]. Initially, the NSM technique was used in Europe to strengthen reinforced concrete structures. Studies [44] were carried out on concrete beams using NSM steel reinforcing bars connected in grooves filled with cement mortar. In addition, studies of the defects and losses of pre-stressing force due to the corrosion of concrete bridge structures have also been carried out [45,46,47]. On the other hand, an experimental study involving the reinforcement of fifteen glulam beams with BFRP bars, GFRP and CFRP cords was described in [48]. The timber members had grooves with dimensions of 15 × 15 mm, and the offset of the grooves from the bottom side of the beam was 33 mm. The two 8 mm diameter CFRP/GFRP rods with epoxy–melamine adhesive were used as the reinforcement for the beams. On the other hand, the larger beam elements were reinforced with three 8 mm diameter rods composed of GFRP/GFRP and glued with epoxy resin; they were then subjected to a four-point bending test. It was observed that the use of FRP reinforcing bars resulted in an increase in bending resistance and ductility. The increased load-bearing capacity was also acquired by timber elements that were strengthened using BFRP bars [42]. Subsequently, studies of timber beams with basalt bars revealed that unbonded BFRP bars had an increased load-carrying capacity and stiffness of 28% and 8.7%, respectively [49]. On the other hand, the beams with bonded reinforcements showed an increased load carrying capacity and stiffness of 15.4% and 11.5%, respectively, compared to the unbonded reinforcing bars.

The transfer of stresses for the NSM method can be described as a chemical adhesion [1] and it occurs at two interfacial zones. At first, the stress transfer occurs at the interface between the reinforcing bar and the adhesive. The second interaction transfers the stresses along the joint between the adhesive and the host wood. On the basis of experimental studies, it has been found that surface roughness, groove dimensions or adhesive behaviour affect the capacity of this bonding. According to the study by De Lorenzis [50], it was observed that in case of a round bars, the width of the groove to the nominal diameter ratio of the bar should be 1.5 for smooth or lightly sand-blasted bars, and that this value should be 2.0 for deformed bars. In addition, the BFRP bars, similar to all other fibre composite materials, exhibit orthotropic mechanical behaviour. Consequently, better performance is obtained when the load is applied in the direction of the fibres, while the strength is usually much lower when the load is applied perpendicularly to the fibres [41]. BFRP bars are, therefore, more susceptible to failure in the anchorage area, due to the wedge devices used for steel tendons, where strong lateral pressures can occur. This liability, however, applies for all types of FRP products. To overcome this disadvantage of FRP materials, various types of anchorage devices are being developed [51,52].

In conclusion, as the research on strengthened timber beams with pre-stressed BFRP bars is still limited, the main objective of this analysis is to verify the effectiveness of strengthening glued laminated timber beams with pre-stressed BFRP rebars and to determine the behaviour of the strengthened beams under static loading for comparison with a numerical model. This paper presents the results of the distribution of stresses, both in the base and strengthening material, in load displacement behaviour, in a comparison between the tested specimens and the numerical model, and also in a description and photos of the failure modes of the specimens. However, it has to be mentioned that, although the long-term behaviour of BFRP reinforcement when exposed to various conditions [53] is a primary concern, especially for pre-stressed members due to pre-stress losses [54,55], this study only provides an insight into resistance and behaviour under short-term loading effects.

## 2. Materials and Methods

### 2.1. Materials

For the strengthening of the glued laminated timber beams, the BFRP bars were used together with an epoxy adhesive. The glued laminated timber beams were characterised as class GL24c, according to PN-EN 14080:2013-07 [56]—Scots pine (*Pinus sylvestris* L.), originating from the Lesser Poland Landscape Nature and Forestry Region of Poland (Table 1).

After slowly drying the wood to an air-dry state, standard samples (mainly heartwood) were cut for the determination of individual physical and mechanical properties. Wood samples of Scots pine (*Pinus sylvestris* L.), from the Kraina B Malopolska Nature and Forestry Region (Nadleśnictwo Przedbórz), were used for the study.

The outer layers of the lamellae were characterised by a T14 grade and the inner layers were characterised by a T9 grade. The grades of the lamellas were determined by strength classification using a visual method, in accordance with PN-D-94021:2013-10 [57] and PN-EN 338:2016-06 [58]. The classified lamellas were glued together to form glued laminated timber beams, and their dimensions were prepared for experimental four-point bending tests, in accordance with PN-EN 408 + A1:2012 [59] and PN-EN 1995-1-1:2010 [60]. To bond the lamellas together, the D4 polyacetylvinyl glue was used (density approx. 1.10 g/cm^3^, viscosity 13,000 mPa.s). The beam elements had a rectangle cross-section with dimensions of 82 × 162 mm and a length of 3650 mm. The average density was 400 kg/m^3^, while the average moisture content ranged from 10.5 to 14.1%.

A two-component epoxy adhesive with a thickness of 2 mm between the wooden component and the BFRP bars was used in this study. The diameter of the rebars was approximately 10 mm. The rebars were produced by the manufacturer Polprek using the pultrusion method. The modulus of elasticity of the rebars used in this study was E = 56.3 GPa, the tensile strength was f_fu_ = 1455.3 MPa, and the elongation at rupture was ε_fu_ = 2.12%, respectively. The epoxy adhesive was used in the tests as filler for the NSM reinforcement. Such bonding is characterised by a favourable bond quality in the interfacial areas, resulting in both higher performance and more favourable gap-filling properties [1]. It should be also taken into account that the two-component epoxy adhesives have better shrinkage and thixotropic properties. The adhesive layer was obtained by mixing LG 385 epoxy resin (density 1.18 ÷ 1.23 g/cm^3^, viscosity 600 ÷ 900 mPa.s) with HG 385 hardener (density 0.94 g/cm^3^, viscosity 50 ÷ 100 mPa.s). After mixing the resin and hardener, the adhesive achieved a flexural strength of 110 ÷ 120 MPa and an elastic modulus of 2700 ÷ 3300 MPa. The epoxy adhesive (LG 385, HG 385) consisted of two parts, which were mixed at a ratio of 2:1 by volume, as required by the product standard.

**Figure 1:**
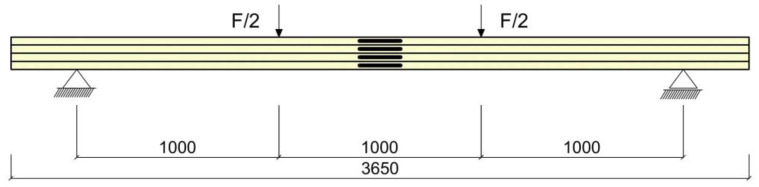
Load case diagram.

**Figure 2:**
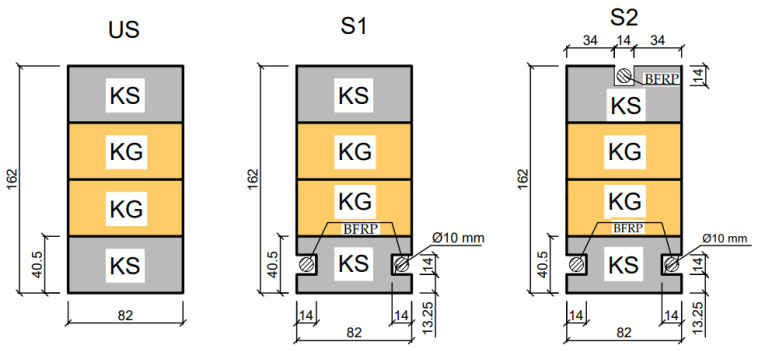
The configuration of the strengthened beams (US—unstrengthened beams; S1—beams strengthened with two pre-stressed BFRP bars in the tension zone; S2—beams strengthened with pre-stressed BFRP bars in the tension and compression zones; KG—class of inferior quality structural timber; and KS—class of medium quality structural timber).

The aim of the study was to determine the ultimate load, strains under the loading, and the modulus of elasticity of the wood in bending, which was carried out in accordance with EN 408 + A1:2012 [59]. The modulus of elasticity in bending was determined as a result of experimental tests performed on unreinforced beams. In total, 15 glued laminated timber beams, including 5 unreinforced beams (US) and 10 reinforced beams (S1 and S2), were prepared for the study. Figure 1 illustrates the configuration of the four-point bending test. The designations of the glued laminated beams are given in Figure 2.

As has already been stated, a total of 15 timber beams were tested in a four-point bending test until failure in this experimental study. The length of all the beams was 3.65 m and they had a rectangular cross-section with dimensions of 82 × 162 mm. The two reinforcement configurations were investigated.

### 2.2. Methods

In the first reinforcement configuration, the tension zone of the beams was reinforced with two BFRP bars (percentage of reinforcement was equal to 1.2%), while in the second configuration, an additional bar was added in the compression zone (percentage of reinforcement equal to 1.9%). Five unreinforced timber (US) beams were additionally tested for comparison purposes. It should be noted that both series of tested reinforcement configurations consisted of 5 specimens. As can be seen from the Figure 1, the length of the span between the supports was 3000 mm. The configurations of the tested beams and the pattern of reinforcement are shown in Figure 2. All dimensions presented in the figures are given in millimetres.

**Figure 3:**
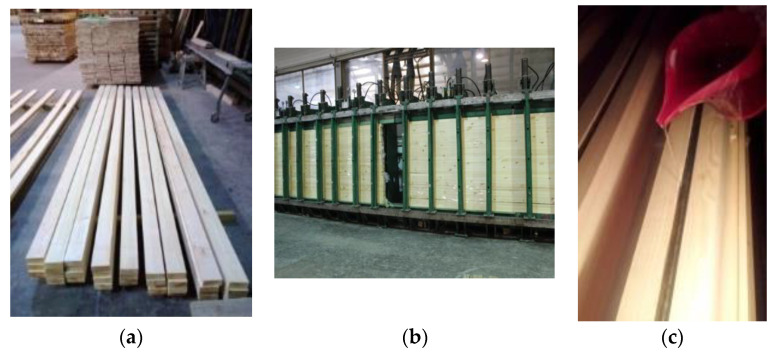
The preparation of the reinforcement of timber beams with BFRP bars: (**a**) strength classification of the sawn timber using a visual method; (**b**) gluing the lamellas together in a hydraulic glulam press; (**c**) application of epoxy resin into the milled grooves, followed by the insertion of basalt bars into the milled grooves and the preparation of pre-stressing. The initial pre-stressing force was 10 kN.

The process of strengthening the timber beams can be seen in Figure 3. Regarding the final position of the reinforcement in the timber beams, the square longitudinal grooves were initially prepared at the considered locations. After that, the grooves were thoroughly cleaned before the bonding process. Finally, the BFRP bars were inserted into the grooves sealed with epoxy resin. Moreover, to ensure the accurate placement and to secure the sufficient bonding between the glued elements, the pressure was applied along the entire length of the reinforced beams. In case of every specimen, the bonding process initially started in the tension zone; meanwhile, the bar in the compression zone was glued 24 h later. The specimens were tested 7 days after the placement of the last bar.

**Figure 4:**
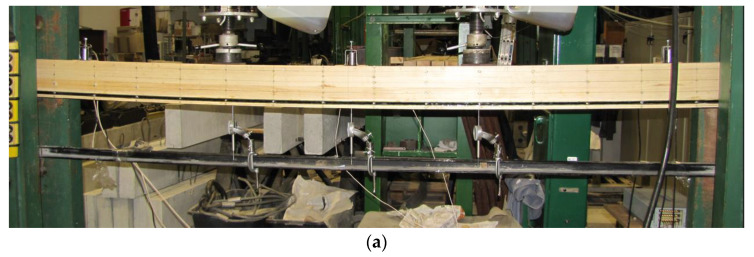

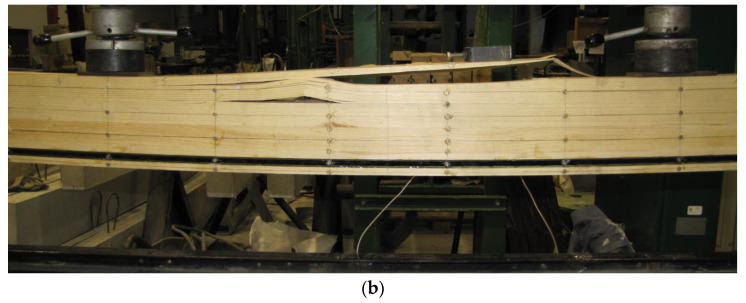
The test set-up of timber beams with BFRP bars: (**a**) strengthened timber beam during loading; (**b**) strengthened timber beam after failure.

The beams were loaded with two hydraulic jacks with a piston area of 50 cm^2^ and a maximum exerted pressure of 10 MPa from the producer VEB Werkstoffprufmaschinen in Leipzig. The type of loading was quasi-static monotonic loading, while the following variables were measured: longitudinal strains—reading deflection values with an 8-inch Demec mechanical extensometer; vertical displacement—reading deflection values with dial gauges; and the breaking load until failure. A total of 6 measuring points were placed linearly along the span to determine the strain distribution. The bases of the mechanical extensometer were placed at the height of the section at the distances of 20.25 mm, 60.75 mm, 74.375 mm and 81 mm from the centre of the section height. On the other hand, the dial gauges were placed in the middle of the span and along the length of 5 h; this includes h, which is the height of the beam cross-section. Load-bearing plates were placed at the support faces to allow load distribution and avoid local stress concentrations. The experimental test setup is presented in Figure 4.

## 3. Results and Discussion

All the results gathered during the experimental analysis are analysed and discussed below. The failure loads and stiffness of the BFRP-reinforced beams were compared with those of the control beams in order to determine the effectiveness of the beam strengthening. In addition, all failure modes are defined below.

### 3.1. Failure Damage Images of Glued Laminated Beams

**Figure 5:**
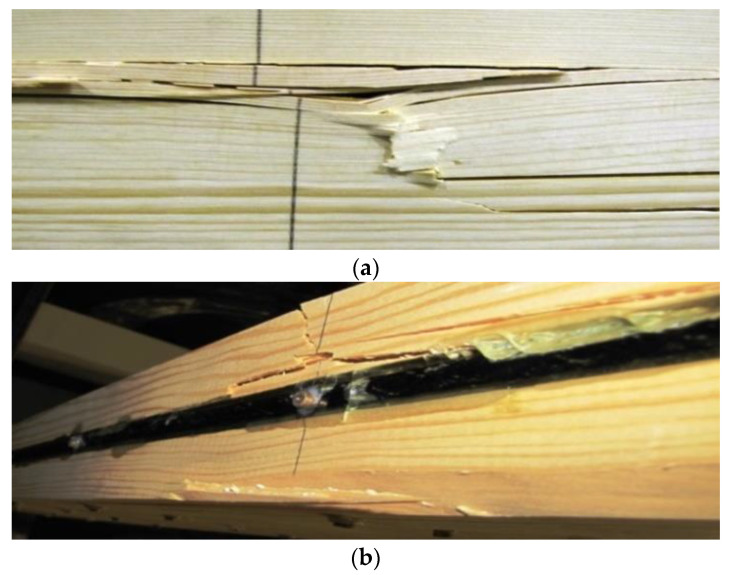
Failure damage images in glued laminated beams strengthened in the tension and compression zones—S2: (**a**) compression zone shear (60 kN); (**b**) wood and epoxy glue detachment in tension zone.

The failure modes of the unreinforced and reinforced timber beams were observed visually (see Table 2). All failure modes were observed and recorded. According to the results of the experimental analysis, it can be seen that the predominant failure mode for both the reinforced and unreinforced beams was failure in the tension zone or crushing in the compression zone, usually at the locations of knots. Furthermore, it was observed that none of the FRP bars failed. The typical failure modes for S2 reinforced beams are shown in Figure 5. 

In the case of the unreinforced beams, they mostly showed deterioration in the tension zone in the presence of defects, including knots. The third reinforced beam from the second series (S23) showed tensile failure, with cracks in the compression and tension zone. It is noteworthy that for the laminated timber beams, the knots were located at the extreme fibres both in the compression and tension zones. The presence of defects in the wood was the most common reason for the premature failure of the beam. Small cracks also developed in the tensioned fibres while the glue next to the reinforcement bars was still intact. There were also kink bands in the compression zone when the beam had reached its maximum load-bearing capacity. In another case for the beam S12, a large knot was visible at the mid-span as a timber structural defect. This was followed by the detachment of the epoxy adhesive from the rebar after cracks had developed in the tension zone.

In the strengthened beams, cracks and propagation around the knots were observed. Moreover, peeling between the bars and the timber was observed in the tension zone of the beams when the failure load was reached (beams S15, S24).

### 3.2. Load-Deflection Curves

**Figure 6:**
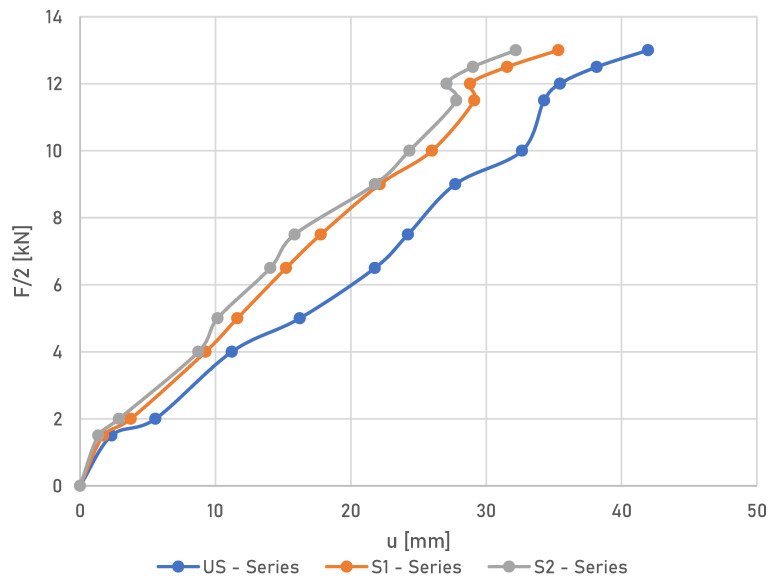
Mid-span load displacement curves averaged for all series.

Figure 6 shows the average load vs. the vertical displacement curves at mid-span for all tested laminated timber beams. It has to be stated that the displacements were measured by mechanical dial gauges; therefore, the measurements were interrupted at the same load due to safety concerns.

As can be observed in Figure 6, the beams showed almost linear load-deflection behaviour until failure. It can also be seen that the failure load of the reinforced beams was significantly higher than the failure load of the unreinforced beams. In addition, the stiffness increased for reinforced beams. In particular, the increase in stiffness for reinforced beams of the S1 series was 16%, and the stiffness of the reinforced beams of the S2 series increased by 23% compared to the unreinforced US beams. In comparison, with a monotonic load of F/2 equal to 12 kN, the deflections for the US beams were 35.45 mm, those for the S1 beams were equal to 28.81 mm and those for the S2 beams were 27.07 mm, respectively. The readout failure load for the S1 beam series was approximately 24.54% higher than for the US beam series. In contrast, for the S2 beam series, the failure load was 47.22% higher with respect to the unreinforced beams of the US series. In some individual cases, this increase was lower due to the presence of hidden defects in the wood, which caused damage to the timber before its full resistance capacity was even reached; meanwhile, the FRP bars limited crack propagation and reduced the destructive impact of the timber defects.

### 3.3. Stress Distribution of Wood and BFRP

**Figure 7:**
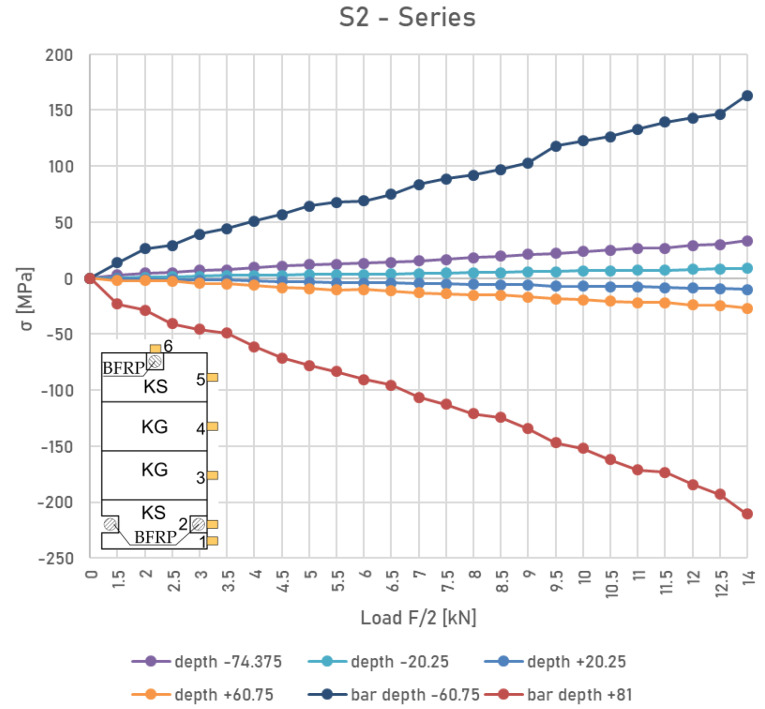
Typical stress distribution profile in the tension and compression zones for S2 series of reinforced beams.

Figure 7 shows a plot of the load versus stress diagram at various fibres across the depth of the cross-section for the S2 series.

It was found experimentally that the linear elastic principles in the initial phase of the study were retained. For some beams reinforced with bonded BFRP bars, the neutral axis is slightly lowered. Furthermore, it was observed that there was a non-linear behaviour in the compression zone compared to the other reinforced beams tested in this experimental study. The stresses in the wood in the most tensioned lamellas at force F/2 = 12 kN were as follows: for the series US—39.22 MPa; for series S1—34.78 MPa; and for series S2—29.25 MPa. On the contrary, the stresses in the most compressed lamellas at force F/2 = 12 kN were as follows: for series US—(−35.14) MPa; for series S1—(−29.33 MPa); and for series S2—(−23.86) MPa, respectively. Regarding the stresses in the bonded strengthening bars, the stresses in the tensioned bars at force F/2 = 12 kN were as follows: for series S1—151.22 MPa and for series S2—143.25 MPa. The stresses in the compressed bars at the same load for the S2 series were −184.25 MPa, respectively.

According to the experimental tests, it was observed that in some cases, cracks appeared in the knot present in the tension zone of the beam as the load increased. Then, as the displacement increased, the cracks were visible in the loading contact area and the crack propagation in the compression zone was limited, even at higher loads, due to the presence of non-linear behaviour in this zone.

### 3.4. The Numerical Analysis of Glued Laminated Timber Beams

The numerical analysis of glued laminated timber beams composed of different KG and KS timber quality classes was carried out in the ANSYS 16.0 environment using the Static Structural module. The geometry of the beams, on the other hand, was made in CATIA V5 software, representing a composite consisting of the following components: solid bodies representing supports and points of application for the loading forces, lamellas, reinforcing bars and glue filling the space between the lamella and the strengthening bar. The finite element mesh was generated from hexa and tetragonal elements. The wood lamellas and the supports were modelled as hexagonal elements with a size of 10 mm. The BFRP rods and epoxy adhesive were defined as tetragonal elements with a size of 5 mm. Therefore, the connection between the glue and timber was defined as a contact connection with “bonded” properties. The following parameters were assumed for the analysis: wood quality grade KS: T14 (C24) and wood quality grade KG: T9 (C14), (see Table 3, Table 4 and Table 5). The modulus of elasticity of the BFRP bars was adopted from the results of tensile tests.

**Figure 8:**
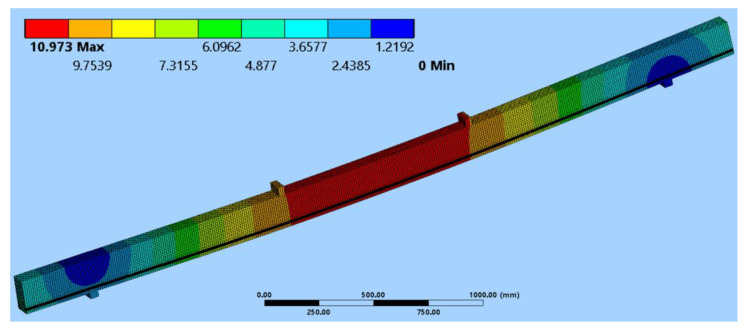
The deflection distribution for the S1 beam series.

In the numerical study, a three-dimensional finite element model was used in order to determine the behaviour of the unreinforced US and reinforced S1 and S2 beams; these were made with the same configuration of timber quality classes, were reinforced with bonded BFRP bars in the tension zone, and then were simultaneously reinforced in both the tension and compression zones, respectively. All of the tested series of beams were also analysed numerically. Both the dimensions of the elements and the static schemes were identical to the laboratory tests. The results from the numerical analysis are presented in the Table 6, while the deflection for the S1 series is presented in Figure 8.

In order to verify the suitability of the design numerical model, a comparison of the results had to be carried out according to the results gathered during the experimental analysis. Therefore, the comparison of the deflections is presented in the Table 6.

It can be seen that the results obtained by the numerical analysis are similar to the experimental results. The slight differences between the results obtained from the numerical analysis and laboratory tests could be a consequence of the simplifications that were applied in the finite element model. It should be remembered that wood is a complex organic material that exhibits anisotropy in terms of mechanical properties. While focusing on the time and on work efficiency when performing numerical calculations, it is impossible to include all the structural complexities of wood (e.g., structural irregularities, wood defects) contained in the specimens, as they are, in most cases, difficult even to determine. Moreover, it is advisable that material tests are carried out to determine the exact values of the mechanical properties for each direction of structural timber.

## 4. Conclusions

This paper presents the results of an experimental study on laminated timber beams reinforced with BFRP pre-stressed bars using the NSM reinforcement technique. Three series of beams, each consisting of five reinforced or unreinforced beams, were tested under quasi-static monotonic loading until failure. These tests yielded the following conclusions:

(1) The strengthening of timber beams with NSM BFRP bars is an effective method of increasing both the stiffness and the load-bearing capacity of bending timber members. The increase in the ultimate load was 35.88%, while the increase in the stiffness was by up to 23% compared to the unreinforced beams.
(2) It was observed that laminated beams reinforced with bonded BFRP bars both in the tension (with two FRP bars) and compression zones (with one FRP bar) showed a higher increase in the ultimate load capacity, compared to when strengthening was only employed in the tension zone.
(3) The most common failure mode was, for both unreinforced beams and reinforced beams using the NSM BFRP techniques, fibre cracking at wood defects, including the knots in the tension zone. Moreover, it should be noted that some beams showed signs of cracking at the epoxy glue joint.
(4) The results obtained with the finite element method are similar to the experimental results. The slight differences found could be the result of the simplifications used in the numerical model or, on the other hand, the hidden defects of the wood that, for obvious reasons, cannot be included in the numerical simulation.

After the experimental studies, it was concluded that further laboratory and numerical studies of NSM FRP-reinforced timber beams are needed due to the heterogeneity of this material; they should also be modelled in different configurations in order to determine the various factors that affect the resistance of timber structures.

## Figures and Tables

**Table 1:** The mechanical properties of glued laminated beams.

Material	Tensile Strength (N/mm^2^)	Compressive Strength (N/mm^2^)	Bending Strength (N/mm^2^)	Shear Strength (N/mm^2^)	Modulus of Elasticity (N/mm^2^)
Timber	17	21.5	24	3.5	11,000

**Table 2:** The ultimate resistance and failure modes of tested beams.

The Failure Load F [kN]	Beam	Average Resistance	Description of the Damage
44	US1	43.2	Shearing of the adhesive bond in the tensile zone and subsequent displacement of the tensile lamellas.
39	US2		The failure occurred at the site of a hidden wood defect—a knot.
47	US3		Delamination of the tensioned lamella at a force of 47 kN, then shearing of the wood along the fibres.
45	US4		Damage to the tension lamellae through the fracture of the tension lumber fibres.
41	US5		Tearing of the wood fibres appeared in the tension lamella, followed by failure in the compression zone due to the present knot.
The failure load F [kN]	Beam	Resistance increase [%]	Description of the damage
51	S11	18.1	The failure occurred initially in the area of the defect—a knot, at the centre of the beam span.
55	S12	27.3	A hidden defect—a knot—appeared in the middle of the span. This was followed by the detachment of the epoxy glue from the rebar.
55	S13	27.3	The failure occurred initially in the area of the defect—a knot, at the centre of the beam span.
57	S14	31.9	In the tensile zone, a shear of the glue joint appeared at first, followed by a displacement of the tensile lamella.
51	S15	18.1	Peeling was observed between the bars and the timber in the tensile zone of the beams when the failure load was reached.
63	S21	45.8	Damage in the tensile zone in place of a hidden defect (knot), followed by crushing in the compression zone.
67	S22	55.1	Separation of epoxy adhesive from the wood occurred in the vicinity of the knot of the tension zone, followed by crushing in the compression zone.
60	S23	38.9	The failure was due to fracture in the compression and tension zones.
64	S24	48.2	Cracking of the wood fibres in the tension zone, then compression. Peeling was observed between the bars and the wood.
64	S25	48.2	Shearing of the compression zone, followed by spalling of the wood and epoxy glue in the tension zone.

**Table 3:** Timber material data.

Type of Wood	Young’s Modulus (MPa)			Poisson’s Ratio			Shear Modulus (MPa)		
	X	Y	Z	X	Y	Z	XY	YZ	XZ
Lamella KS	11,000	363	363	0.54	0.027	0.54	690	69	690
Lamella KG	7000	230	230	0.54	0.027	0.54	440	44	440

**Table 4:** The material data of BFRP bars.

Type of Bar	Young’s Modulus (MPa)	Tensile Strength (MPa)	Compressive Strength (MPa)	Elongation at Rupture [%]
BFRP	56,300	1455.3	945.9	2.12

**Table 5:** The material data of the epoxy adhesive.

Type of Material	Young’s Modulus (MPa)	Tensile Strength (MPa)	Compressive Strength (MPa)	Elongation at Rupture [%]
Epoxy glue	3000	80	140	6

**Table 6:** A comparison of deflection values using laboratory and numerical methods, for the force value F/2 = 5 kN.

Series	Experimental Analysis—Mean Value	Numerical Analysis	Difference
	u [mm]	u [mm]	[%]
US	16.23	15.12	6.84
S1	11.62	10.97	5.59
S2	10.16	9.67	4.82

## Data Availability

Not applicable.
